# Machine learning-based transcriptomic analysis identifies NAMPT and SAT1 as potential biomarkers and therapeutic targets in ferroptosis-associated rheumatoid arthritis

**DOI:** 10.1371/journal.pone.0333556

**Published:** 2025-09-29

**Authors:** Devi Soorya Narayana Sasikumar, Vino Sundararajan

**Affiliations:** 1 Integrative Multiomics Lab, School of Bio Sciences and Technology, Vellore Institute of Technology, Vellore, Tamil Nadu, India; Central South University, CHINA

## Abstract

**Background:**

Rheumatoid arthritis (RA) is an autoimmune disease with chronic presentation, involving symmetric joints and systemic involvement. Ferroptosis is iron-dependent programmed cell death through lipid peroxide accumulation, implicated in inflammatory diseases, including RA. However, its underlying mechanisms and gene-level contributions to RA pathogenesis remain largely unexplored. Therefore, this study emphasizes identifying ferroptosis-related genes associated with RA, evaluating their diagnostic, prognostic, and therapeutic potential, and exploring their role in immune modulation.

**Methods:**

The transcriptomic dataset (GSE89408) from the peripheral blood gene expression was downloaded from the Gene Expression Omnibus (GEO) database. We extracted the differentially expressed genes (DEGs) using R software and the most relevant modules relevant to RA were identified through weighted gene coexpression network analysis (WGCNA). We also identified the differentially expressed ferroptosis genes. The gene ontology and pathways involving the common genes were identified and the protein-protein interaction network was constructed. The hub genes were identified using three machine learning algorithms, least absolute shrinkage and selection operator (LASSO), random forest (RF), and support vector machine (SVM), after which the diagnostic efficiency of the hub genes and the correlation with immune infiltrating cells were predicted.

**Results:**

A total of 9176 DEGs and a module of 314 genes were obtained which has a significant correlation with RA and 17 genes were selected after the intersection. Using the three machine-learning algorithms, we retrieved 8 hub genes (CISD2, LACTB, PRNP, SAT1, NAMPT, MITD1, SOD2, and FASN) between RA and ferroptosis which showed good diagnostic performance using the ROC curve and nomogram plots. Functional annotation analysis was utilized to inspect the biological functions of the hub genes and the genes showed a substantial association with the immune infiltrating cells.

**Conclusion:**

NAMPT, CISD2, LACTB, PRNP, SAT1, SOD2, MITD1, and FASN may modulate ferroptosis and RA by influencing immunity, and NAMPT and SAT1 contribute significantly to the diagnosis and treatment of the disease. Future studies focusing on validating these genes in larger cohorts and exploring their therapeutic potential will provide deeper insights.

## 1. Introduction

Rheumatoid arthritis (RA) is a persistent autoimmune disorder predominantly targeting the joints, marked by chronic inflammation of the bilateral joints, damaging the joint synovium, advancing to cartilage destruction, bone erosion, and impaired function of the joints [1]. Beyond its local effects, RA has been reported to exert systemic manifestations throughout the body [2,3]. Histological examinations of the RA-affected tissues reveal notable synovial membrane hyperplasia, accompanied by increased vascularity of the blood vessels within the synovial membrane [4]. This enhanced vascularity facilitates immune cell infiltration, particularly T-cells, B-cells, and macrophages, into the joint cavity, resulting in the formation of pannus tissue, which invades the joint cartilage and bone, ultimately culminating in bone erosion and structural damage to the joints [5,6].

Programmed cell death, including apoptosis, autophagy, pyroptosis, necroptosis, and ferroptosis, plays a pivotal role in the pathogenesis of RA. An imbalance in Programmed cell death may cause a variety of immune cells to release large amounts of inflammatory factors and mediators that exacerbate not only chronic synovial inflammation, but also bone and joint damage [7]. Diverging from the traditional programmed cell death, like apoptosis or necrosis, in both mechanism and cell morphology, ferroptosis represents a pronounced form of regulated cell death propelled by iron-dependent lipid peroxidation [8]. A differentiating characteristic of ferroptosis is the inactivation of glutathione peroxidase 4 (GPX4) activity, leading to the accumulation of toxic lipid peroxides [9,10]. Notably, ferroptosis differs from other cell death mechanisms as it does not involve DNA fragmentation or membrane blebbing; instead, it leads to cell death through mitochondrial shrinkage and increased membrane density [9]. Ferroptosis occurs mostly in those tissues with elevated oxidative stress and iron concentrations and has been strongly implicated in diseases such as neurodegeneration, inflammatory bowel disease, various cancers, and RA [11–13]. Mitochondrial abnormalities, lipid peroxidation markers, and disrupted iron homeostasis are the major histological features that signal the cellular integrity breakdown, ultimately contributing to systemic tissue damage [14,15].

RA and ferroptosis exhibit fascinating interconnections through their association with oxidative stress, chronic inflammation, and iron dysregulation, all of which synchronously contribute to tissue damage in RA and are central to the course of ferroptosis [16]. The evidence from recent studies suggests that ferroptosis is instrumental in the pathological progression of RA, by promoting joint destruction through lipid peroxidation and inflammatory cascades within the synovium [17–19]. Although no specific inhibitors have been developed to target RA and ferroptosis simultaneously, antioxidants are of growing interest due to their potential dual therapeutic effects. However, inhibitors like ferrostatin-1 specifically target ferroptosis and have shown the potential to reduce the damage caused by oxidative stress [20]. Given the therapeutic potential of targeting ferroptosis to extenuate RA progression, further studies are imperative to augment our understanding of its role in disease pathogenesis and to identify biomarkers that link ferroptosis and RA, which might facilitate the identification of novel therapeutic targets and strategies.

In this study, we inspected the role of ferroptosis-related genes in RA by analyzing the transcriptomic dataset (GSE89408) obtained from GEO. DEGs and the highly associated gene modules were identified using R software, and the common genes were subjected to gene ontology and pathway analysis. We applied machine learning algorithms, including LASSO, random forest (RF), and support vector machine-recursive feature elimination (SVM-RFE), to identify the key genes, discern the hub genes, assess their diagnostic potential, and correlate them with immune cell infiltration patterns. This study aims to elucidate the role of ferroptosis in RA, identify robust biomarkers for early diagnosis and prognosis, and create a prediction model.

## 2. Materials and methods

### 2.1. Data collection

The bulk RNA dataset GSE89408 was retrieved from the GEO database (https://www.ncbi.nlm.nih.gov/geo/), based on the GPL11154 Illumina HiSeq 2000 platform. This dataset comprised 218 samples, including 152 RA (46 males and 106 females) samples and 28 healthy control tissue samples (14 males and 14 females) from the synovium, which were utilized further for this study [21]. The dataset GSE89408 includes only treatment-naïve RA patients, reducing confounding due to medication effects. While RA samples are annotated as ‘early RA’ and ‘established RA’, detailed clinical staging criteria were not uniformly available in the metadata; therefore, all RA samples were analyzed together as a unified group. Additionally, ferroptosis-associated genes were curated from the GeneCards database (https://www.genecards.org/) and the relevant literature search [22].

### 2.2. DEG identification

The DEGs were identified from the normalized expression matrix of the dataset utilizing the ‘DESeq2’ package, with the screening criteria set at a *p-*value ≤ 0.05 and the |log2 fold change (logFC)| > 1 [23]. To visualize the results, a heatmap was generated using the ‘pheatmap’ package, while the ‘EnhancedVolcano’ and ‘ggplot2’ packages were employed to construct a volcano plot highlighting the upregulated and downregulated genes [24].

### 2.3. Identification of the key module genes

WGCNA is a systems biology approach designed to characterize gene association patterns across large datasets [25]. Prior to network construction, we performed hierarchical clustering of samples to detect potential outliers based on sample connectivity. Utilizing the normalized expression data, the soft-thresholding power was calculated based on the scale-free network topology criterion. A minimum module size of 30 genes was established during the dynamic tree-cut method to ensure robust module detection [25,26]. The module exhibiting the strongest correlation with RA was identified, and the module membership, as well as gene significance, were subsequently determined.

### 2.4. Functional enrichment analysis and PPI network construction

To find the differentially expressed ferroptosis genes (DEFeGs), the intersection of the DEGs and ferroptosis-associated genes was determined. Additionally, the overlap between significant module genes and the DEFeGs was analyzed to identify the shared genes. To inspect the biological function of these common genes linked to ferroptosis and RA, gene ontology (GO) and pathway enrichment analyses were conducted using the DAVID web-based server (https://davidbioinformatics.nih.gov/), which computes statistical significance using a modified Fisher’s exact test (EASE score) to identify overrepresented pathways and biological processes. The interactions with medium confidence between these common proteins were searched using the Search Tool for the Retrieval of Interacting Genes (STRING) database (https://string-db.org/), and a protein-protein interaction network was created, which was visualized and analyzed using Cytoscape, generating a network representation [27].

### 2.5. Feature selection with machine learning

Machine learning algorithms are powerful methods that can enhance the accuracy of gene selection. To achieve this objective, we used the Least Absolute Shrinkage and Selection Operator (LASSO), Random Forest (RF), and Support Vector Machine (SVM) algorithms for feature selection, which are well-suited to handle the high-dimensional gene expression data. LASSO performs feature selection by shrinking the coefficients of less relevant features to zero, to reduce the overfitting, and is useful for datasets with limited samples and a considerable number of predictors [28]. RF ranks the genes based on the contribution to the accuracy of classification across various decision trees, handling non-linear relationships, and overfitting issues [29]. In contrast, SVM is an influential classifier that identifies genes by constructing an optimal hyperplane to separate different sample groups [30]. These models were implemented using the linear_model, ensemble, and svm modules of the scikit-learn Python library, specifically employing the LassoCV, Lasso, RandomForestClassifier, and SVC classes.

Initially, the dataset was divided into two segments: training and test sets, in a ratio of 70:30. Before training the ML models, the training set was subjected to Stratified K-Fold Cross-Validation, while the test set remained completely deferred. This stratified approach ensures that each fold maintains the original class distribution (RA vs. control), which is particularly crucial given the dataset imbalance. Stratified K-Fold Cross-Validation is a robust method for evaluating the effectiveness and generalizability of ML models [31]. The training data is split into k equally sized folds, where the proportion of classes is preserved within each fold. Iteratively, the model is trained on k-1 folds and evaluated on the remaining fold. This process is repeated k times, ensuring each fold serves as the validation set exactly once. The performance of the model is averaged across all iterations to achieve a stable performance estimate.

Additionally, GridSearchCV was employed during the cross-validation process to perform comprehensive hyperparameter tuning [31]. It tests multiple combinations of hyperparameters and selects the best-performing model configuration based on cross-validation scores. This essentially prevents data leakage, where information from the test set influences model training, leading to inflated performance metrics. The cross-validation and hyperparameter optimization steps were implemented using the StratifiedKFold and GridSearchCV classes from the scikit-learn Python library.

The dataset (GSE89408) used in our study exhibits an imbalance between the number of RA samples (n = 152) and normal control samples (n = 28), which poses a risk of biased model development and reduced sensitivity to the minority class (control). To address this limitation, we applied the Synthetic Minority Oversampling Technique (SMOTE), a widely used resampling strategy designed to balance class distribution by generating synthetic examples of the minority class [32]. SMOTE works by identifying each minority instance’s k-nearest neighbours and interpolating new synthetic samples along the feature space. This oversampling step was essential for improving the classifier’s ability to detect features relevant to the underrepresented control group, thereby enhancing the robustness of downstream gene selection.

To evaluate the performance and generalizability of the machine learning classification models, we used the standard classification metrics such as accuracy, precision, recall, F1 score, and area under the curve (AUC) [33]. These metrics were derived from the confusion matrix, which summarizes true positives (TP), true negatives (TN), false positives (FP), and false negatives (FN). Precision was calculated as the proportion of TP to the sum of TP and FP, reflecting the model’s ability to correctly identify positive instances.


$$Precision=\frac{TP}{TP+FP}$$

Recall, also referred to as sensitivity, was computed as the ratio of TP to the sum of TP and FN, indicating the model’s effectiveness in capturing actual positive cases.


$$Recall=\;\frac{TP}{TP+FN}$$

Accuracy was defined as the proportion of correctly predicted outcomes (TP and TN) over the total number of predictions.


$$Accuracy=\;\frac{TP+TN}{Total\;Predictions}$$

The F1 score, a harmonic mean of precision and recall, was used to provide a balanced assessment, particularly in the presence of class imbalance.


$$F\text{1}\;score=\text{2}*\frac{Precision*Recall}{Precision+Recall}$$

Additionally, the AUC metric was employed to assess the model’s discriminatory ability across various threshold settings. All classification metrics were computed using the ‘sklearn.metrics’ module in Python.

Eventually, the hub genes consistently selected across all three models were selected for further biological investigation, and the disease connections were explored using the GeneCards database (https://www.genecards.org/).

### 2.6. Diagnostic efficacy evaluation and immune infiltration analysis of hub genes

To evaluate the diagnostic efficiency of the hub genes, we constructed a nomogram model using the ‘rms’ package in R. Additionally, the ‘pROC’ package was utilized to assess the feasibility of the hub genes to differentiate RA samples from the control samples [34]. Further, the correlation between these hub genes was analyzed using the ‘corrplot’ package in R [35].

### 2.7. Gene ontology and immune infiltration analysis of the hub genes

To explore the functional annotation of the hub genes obtained, we utilized the gseGO function from the clusterProfiler package in R, in which genes are ranked based on log 2-fold change and p-values, and Gene Set Enrichment Analysis (GSEA) is performed to identify the significantly enriched GO terms using permutation-based p-value estimation and FDR correction. The Human Protein Atlas database (https://www.proteinatlas.org/) was used to assess the immune infiltration patterns associated with the hub genes.

### 2.8. Regulatory element analysis and validation of hub genes

To identify the key regulatory molecules of the hub genes, the transcription factor (TF)- miRNA coregulatory network was constructed and visualized using the Network Analyst database (https://www.networkanalyst.ca/) [36]. Furthermore, to examine the expression variations of these hub genes, we used an external RNA-Seq dataset (GSE90081), and the analysis was carried out using the ‘DESeq2’ package. All statistical analyses were performed in R, with a significance threshold set at p < 0.05.

## 3. Results

### 3.1. Dataset collection and identification of DEGs

**Fig 1:**
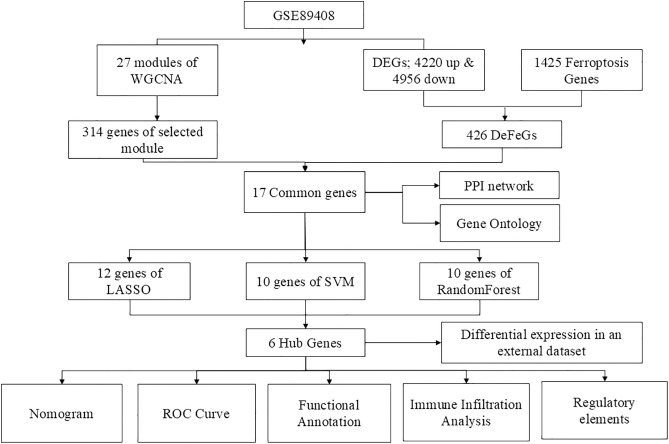
Research process of our study.

**Fig 2:**
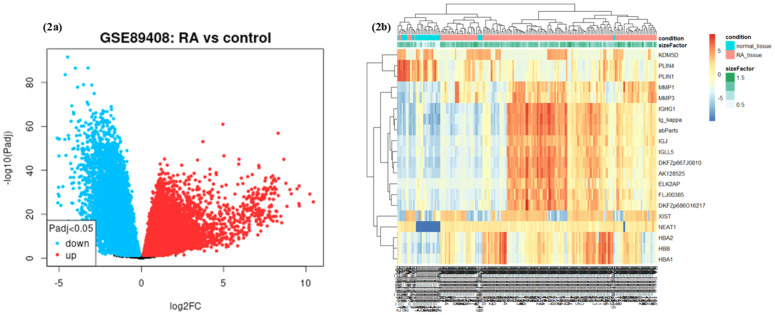
(a) The volcano plot of GSE89408, where the red scatter represents the upregulated genes and the blue scatter represents the downregulated genes. (b) The heatmap of GSE89408.

Fig 1 illustrates the comprehensive workflow of our study. Following the count normalization of the GSE89408 dataset (Additional Figure 1; S1 in S1 File), we identified 9176 differentially expressed genes, consisting of 4220 upregulated and 4956 downregulated genes (Additional File; S2 in S1 File). Fig 2a presents the volcano plot, visualizing the distribution of up- and downregulated genes based on their significance and fold change. The heatmap in Fig 2b displays the expression of the top 20 most differentially expressed genes after normalization. Furthermore, we curated a list of 1425 ferroptosis-related genes from the GeneCards database and relevant literature using the keyword ‘ferroptosis’ (Additional File; S3 in S1 File) [37].

### 3.2. Identification of the key WGCNA modules and extraction of common genes

**Fig 3:**
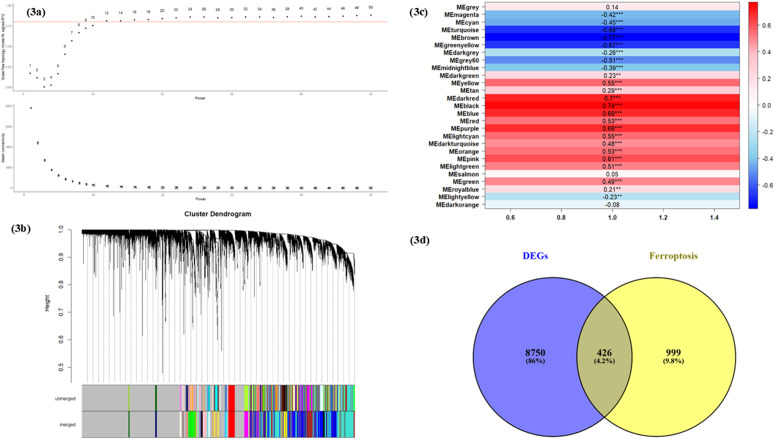
(a) The network topology analysis to identify the optimum soft threshold power. (b) Dendrogram of clustered genes, with each branch representing a gene. The genes clustered into the same module are given the same color. (c) 27 modules with varied colors were obtained, positively and negatively correlated with RA. The black module was positively correlated, having a Pearson correlation coefficient of 0.74, with a p-value < 0.0001. (d) The Venn diagram demonstrates the intersection between DEGs and ferroptosis-associated genes.

**Fig 4:**
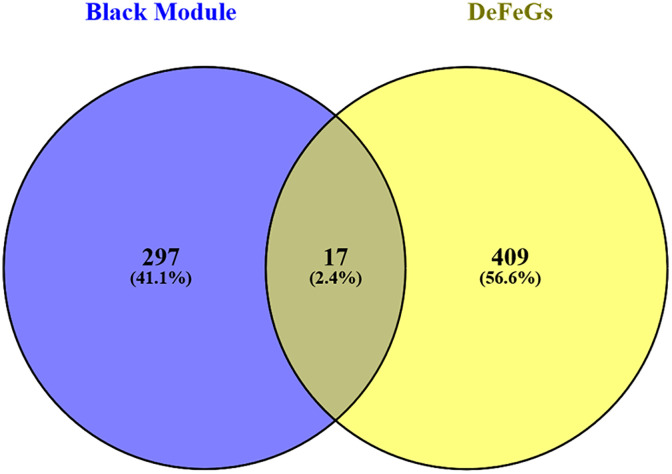
The Venn diagram showing the intersection of common genes between WGCNA and DEFeGs.

To identify the key module genes highly associated with RA, we employed the WGNA algorithm. To attain a scale-free network topology, we selected a soft-power threshold of 12 through trial and error, resulting in an R^2^ value of 0.80 (Fig 3a). Using the dynamic tree-cut algorithm, we obtained 27 key modules based on their correlation with RA, with a minimum module size set to 30 (Fig 3b). These modules include both positively and negatively correlated groups. The positive correlations indicate modules where gene expression levels are elevated in RA samples, suggesting their potential involvement in disease progression, while the negative correlation suggests a protective or regulatory role, where the gene expression is reduced in RA. The MEblack module, which exhibited a strong positive correlation with RA (correlation score = 0.74), highlights its potential as a key player in RA pathogenesis (Fig 3c). Subsequently, we intersected the 9176 DEGs with the 1425 ferroptosis-associated genes to identify 426 differentially expressed ferroptosis genes (DEFeGs) (Fig 3d). Finally, the 426 DEFeGs were intersected with 314 genes from the black module of WGCNA (Additional File; S4 in S1 File) to obtain the final list of 17 common genes associated with both ferroptosis and RA (Fig 4).

### 3.3. Interaction network and functional enrichment analysis

We performed the gene ontology and pathway analysis for the 17 common genes to understand the mechanisms and pathways shared between RA and ferroptosis. The GO analysis revealed several key biological processes significantly enriched in these genes, including the positive regulation of the nitric-oxide synthase biosynthetic process which plays an important role in inflammatory responses, removal of superoxide radicals, which is critical for mitigating oxidative stress, and negative regulation of IL-17 production, a cytokine implicated in RA pathogenesis. Many of these genes were located in the extracellular exosomes, suggesting their potential role in intercellular communication and disease progression. Molecular function analysis showed the genes to be largely enriched in identical protein binding, highlighting the potential protein-protein interactions essential for cellular signalling. Pathway enrichment analysis showed ferroptosis and peroxisome, followed by fatty acid biosynthesis, to play an important role in RA. Ferroptosis, characterized by iron-dependent lipid peroxidation, has the potential to contribute to joint tissue damage; on the other hand, the peroxisome and fatty acid biosynthesis pathways may influence the inflammatory environment in RA. Also, to understand the inter-relations of common genes between RA and ferroptosis, we used the STRING database, extracted the interactions with medium confidence to create a network, and visualized using Cytoscape software, revealing 17 nodes and 13 edges (Additional Figure 2; S5 in S1 File).

### 3.4. Machine learning-based identification of hub genes

Before applying feature selection methods, we performed an oversampling technique like SMOTE, which enhances the model performance by adding data to the minority class to reduce information loss. The classification report was generated for the LASSO, RF, and SVM and is tabulated in Table 1. The LASSO model had an accuracy of 96%, the RF model showed an accuracy of 97.2%, and the SVM model at an accuracy of 97.5%.

**Table 1:** The classification report of the generated LASSO, RF, and SVM models.

Metric	LASSO	Random Forest	SVM
Accuracy	0.960	0.972	0.975
Precision	1.000	0.968	0.958
Recall	0.967	1.000	1.000
Specificity	1.000	0.833	0.750
F1 score	0.983	0.984	0.979
Mean Squared Error	0.053	0.030	0.037
False Positives	0	1	2
False Negatives	1	0	0

**Fig 5:**
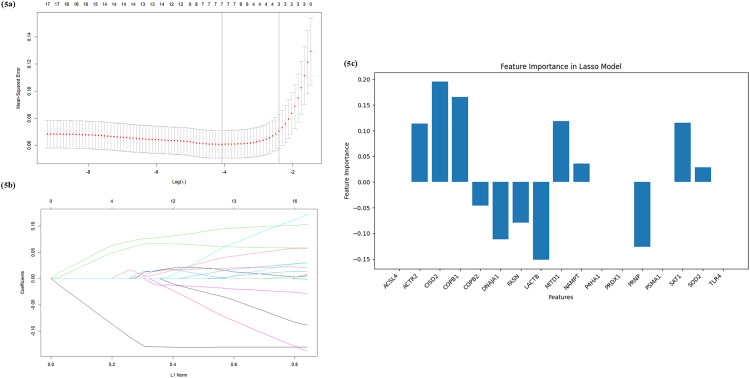
(a-b) The LASSO algorithm was used for hyperparameter tuning and identification of the important predictive features. (c) The top genes chosen by the LASSO model as important features.

**Fig 6:**
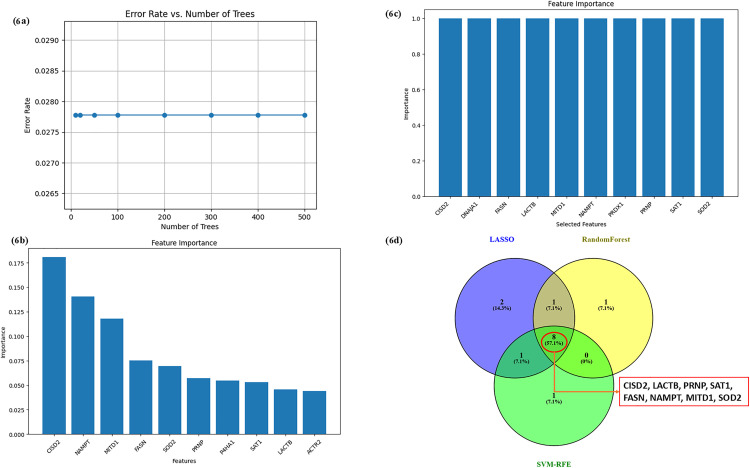
(a) The plot is visualized to understand the association between the number of trees in our RF model and its error rate. The stable error rate with different numbers of trees indicates that the model has an optimal performance level with the current number of trees. (b) The genes that are predicted as hub genes by the RF model. (c) The genes identified by the SVM model as important features. (d) The Venn diagram shows the intersection of features identified by LASSO, RF, and SVM-RFE models.

The 17 common genes identified in the previous step were used to identify the hub genes associated with both ferroptosis and RA by employing three machine learning algorithms- LASSO, RF, and SVM. The LASSO regression algorithm discerned 12 out of the 17 common genes, including ACTR2, CISD2, COPB1, COPB2, DNAJA1, FASN, LACTB, MTD1, NAMPT, PRNP, SAT1, AND SOD2 (Fig 5a–c). The RF algorithm identified CISD2, NAMPT, MITD1, FASN, SOD2, PRNP, P4HA1, SAT1, LACTB, and ACTR2, as the top 10 variables (Fig 6a, b). The SVM algorithm determined CISD2, DNAJA1, FASN, LACTB, MITD1, NAMPT, PRDX1, PRNP, SAT1, and SOD2 as features with equal importance (Fig 6c). By intersecting the top features identified by all three algorithms, we identified the final set of hub genes, which include CISD2, LACTB, PRNP, SAT1, FASN, NAMPT, MITD1, and SOD2 (Fig 6d). These genes were consistently identified across the three approaches, reinforcing their potential role as key players in both ferroptosis and RA, and the functional details of these genes are tabulated in Additional File; S6 in S1 File.

### 3.5. Evaluation of diagnostic performance of hub genes

**Fig 7:**
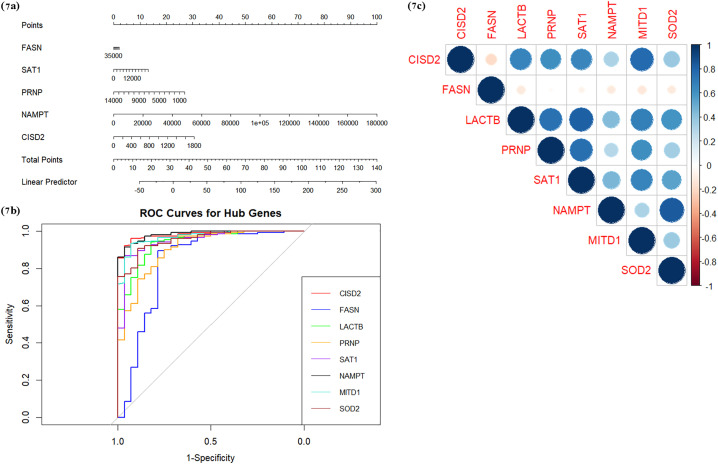
a-c: (a) The nomogram model developed for the prognostic prediction of hub genes from the study. (b) The ROC curves of CISD2, FASN, LACTB, PRNP, SAT1, NAMPT, MITD1, and SOD2 in RA samples. (c) The correlation relationship between the 8 hub genes. The correlation coefficient is labeled by the depth of the color in the chart.

In the multivariate analysis, we employed the Variance Inflation Factor (VIF) to assess multicollinearity, where MITD1, LACTB, and SOD2 exhibited high VIF values of 4.80, 5.55, and 4.87 respectively, indicating high multicollinearity with other predictors in the model. High multicollinearity can distort the parameter estimates, and inflate standard errors, in turn affecting the reliability of the model predictions. To mitigate these issues and improve the accuracy of the model, we excluded MITD1, LACTB, and SOD2 from the further diagnostic performance evaluation. To evaluate the diagnostic potential of the hub genes, we constructed a nomogram based on the remaining 5 hub genes: CISD2, PRNP, SAT1, FASN, and NAMPT. This model allows for the estimation of RA risk by summing the ‘Points’ assigned to each gene yielding the ‘Total Points’ in the nomogram (Fig 7a). Additionally, we conducted a receiver operating characteristic (ROC) analysis to further evaluate the diagnostic relevance of the hub genes. The area under the curve (AUC) values indicated that NAMPT, CISD2, and SAT1 demonstrated the highest diagnostic potential, showing strong discriminatory ability between RA and control samples (Fig 7b). While FASN (72.28% − 94.17%), PRNP (84.72% −96.77%), and LACTB (89.52% −98.29%) also showed good diagnostic value, their confidence interval (CI) ranges indicate slightly less consistency than NAMPT (96.93% − 99.88%), MITD1 (94.48% − 99.52%), SOD2 (92.43% − 98.6%) and SAT1 (90.96% − 99.13%). Finally, the correlation analysis of the hub genes revealed that most of the hub genes were positively correlated with each other, suggesting potential co-regulatory relationships in the context of RA. Only a few genes exhibited negative correlations, highlighting complex interdependencies among the identified biomarkers (Fig 7c).

### 3.6. Functional annotation and immune infiltration analysis of hub genes

**Fig 8:**
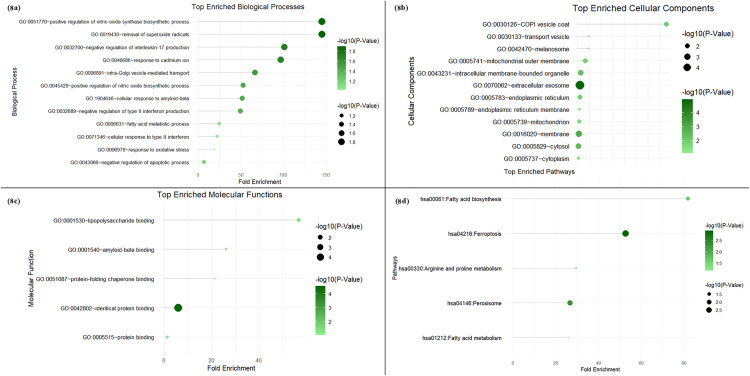
The results of gene ontology analysis of the hub genes, including (a) biological process, (b) cellular components, (c) molecular functions, and (d) pathway analysis.

**Fig 9:**
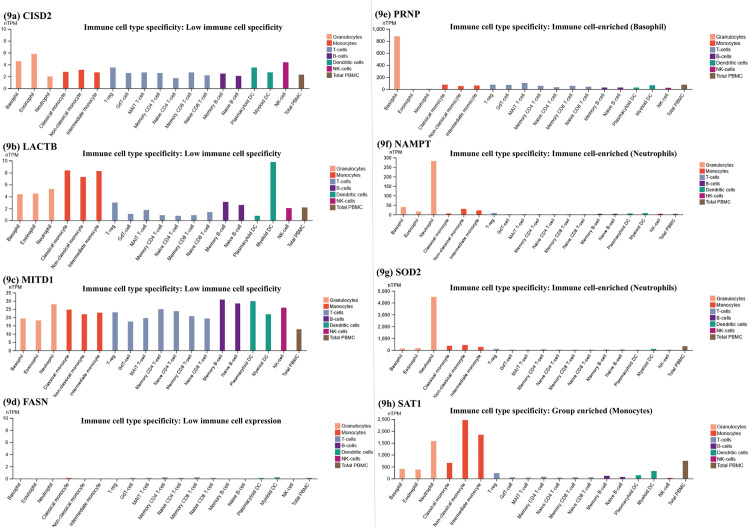
a-h: The expression differences of the 8 hub genes in 18 immune infiltrating cells.

To delineate the ontologies associated with the hub genes, we performed a functional annotation analysis using the clusterProfiler package in R, revealing that CISD2 is mainly associated with the regulation of autophagy, a critical process in cellular homeostasis and immune response, which is known to be altered in RA. LACTB was involved in proteolysis and regulation of the lipid metabolic processes, crucial in maintaining cell membrane integrity and inflammatory pathways in RA. PRNP was linked to the negative regulation of type II interferon, IL-17, and IL-2 production along with negative regulation of the apoptotic process, suggesting its potential role in modulating immune responses and survival of synovial cells in RA. SAT1 strongly influenced angiogenesis which could be important in neovascularization observed in inflamed RA tissues. FASN was implicated in osteoblast, monocyte, and neutrophil differentiation, processes that are involved in bone remodeling, and the inflammatory response in RA. NAMPT revealed its association with signal transduction, cell-cell signaling, and positive regulation of cell population proliferation, which has the potential to contribute to the pathological expansion of immune cells in RA. Another hub gene, MITD1, was associated with mitotic cytokinesis and negative regulation of protein binding, indicating its potential role in cell division and maintenance of cellular structures during inflammation. Finally, SOD2 was involved in response to superoxide, hypoxia, release of cytochrome c, glutathione, and superoxide metabolic process, apoptotic signaling, and negative regulation of fibroblast proliferation, which are key features in RA (Fig 8a–d) (Additional File; S7, S8, S9, S10 in S1 File). Further, the correlation of the hub genes and 18 immune infiltrating cells was evaluated using the Human Protein Atlas database. CISD2, LACTB, and MITD1 revealed a low specificity of immune cell type. CISD2 was enriched across all the immune cell types, including T-cells, B cells, and NK cells; on the other hand, LACTB and MITD1 were enriched largely in innate immune cell types, such as monocytes, dendritic cells, basophils, eosinophils, and neutrophils (Fig 9a–c).

While, FASN showed low immune cell expression with minimal expression observed in memory CD4+ and CD8 + T cells, monocytes, and myeloid dendritic cells (Fig 9d), PRNP showed a preferential enrichment pattern in basophils and NAMPT and SOD2 showed specific enrichment in neutrophils (Fig 9e–g). The SAT1 gene showed group enrichment in monocytes and neutrophils, indicating its expression in a subpopulation of innate immune cells (Fig 9h).

### 3.7. Characterisation of regulatory elements and validation of hub genes

**Fig 10:**
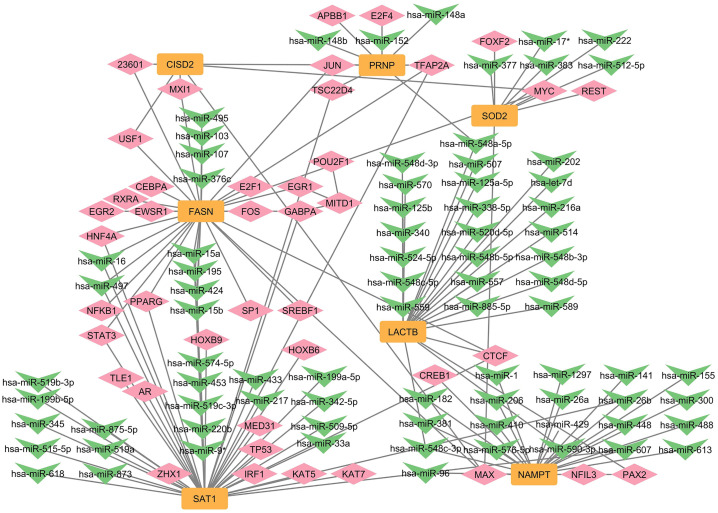
The coregulatory network analysis of the hub genes from the study.

**Fig 11:**
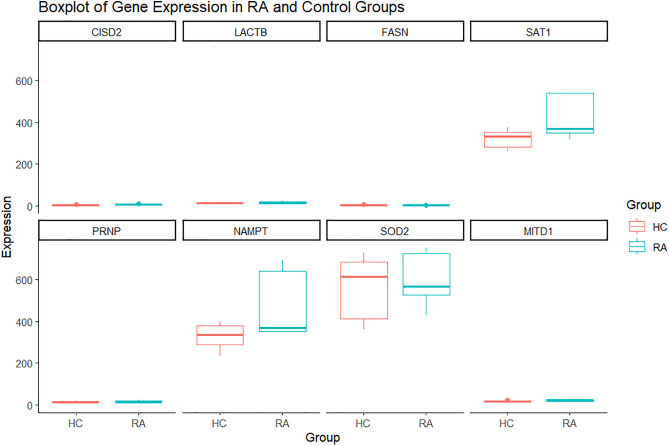
The box plot depicting the differential expression validation of the hub genes in the external dataset GSE90081.

To gain further insights into the regulatory mechanisms underlying the hub genes, we constructed a coregulatory network highlighting interactions between hub genes (orange rectangles), miRNAs (green arrows), and TFs (pink diamonds). The resultant network comprised 128 nodes and 153 edges, showing the complex associations between the regulatory elements (Fig 10). For instance, NAMPT interacts with critical transcriptional factors like CREB1, NFIL3, and hsa-miR1, which regulate immune response and cellular metabolism, potentially linking NAMPT to the inflammatory pathways observed in RA. Another hub gene having diverse connections is SAT1, which interacts with TFs like HOXB6, and TLE1, and miRNAs like has-miR-574-5p, which also interacts with FASN, showing its role in modulating multiple hub genes. MAX, a TF, was identified to have connections with multiple hub genes NAMPT, SOD2, FASN, and CISD2, emphasizing its pivotal role in regulating genes involved in oxidative stress and inflammation. Our study observed that SAT1 and FASN had the most common regulatory elements, indicating that these genes may be coregulated, possibly functioning in complementary pathways. The shared regulation of these genes suggests their potential involvement in closely linked processes like lipid metabolism, cellular stress response, or inflammation. To validate the expression levels of the hub genes identified in our study, we used an external dataset (GSE90081) consisting of RA and healthy control groups, which would strengthen the reliability and robustness of our study findings (Fig 11). The analysis showed that the expression of NAMPT is significantly higher in RA than in the control group, supporting its role as a potential biomarker for RA. A similar trend was observed in the expression of SAT1, indicating its potential as an upregulated gene in RA. Conversely, the SOD2 expression showed that the median expression in control samples was higher than the RA samples, implying downregulation of SOD2 in RA samples, indicating its contribution to increased oxidative stress and inflammation. While FASN showed lower expression in RA, the difference was less pronounced than in the control group, possibly reflecting its more complex role in RA. In contrast, the expression levels of CISD2, LACTB, and PRNP showed minimal difference between RA and the control group, suggesting that their role might be more towards the regulatory mechanisms that are less influenced by disease status. These findings reflect the potential of NAMPT, SAT1, SOD2, and FASN in RA’s pathogenesis, highlighting their potential as biomarkers or therapeutic targets.

## 4. Discussion

RA and ferroptosis share several key pathological mechanisms, including oxidative damage, chronic inflammation, and lipid peroxidation, which are tightly interconnected. In RA, elevated iron levels and the promoters of oxidative stress contribute to ferroptosis, a regulated cell death that leads to synovial cell death and deterioration of cartilage health. This process amplifies the progression of RA, creating a feed-forward loop where the inflammatory microenvironment of RA augments ferroptosis, and, conversely, ferroptosis aggravates inflammation by releasing damage-associated molecular patterns (DAMPs). These DAMPs further activate immune cells, perpetuating inflammation and tissue destruction. The dysregulation of the genes associated with ferroptosis in RA may influence the activation of the immune cells leading to the progression of the disease. Specifically, targeting the ferroptosis-related pathways could modulate inflammation and prevent further joint damage in RA. Building on existing studies, our research aims to investigate the role of ferroptosis-associated genes in RA pathogenesis, and their potential to be a diagnostic/ prognostic biomarker and act as a therapeutic target.

We identified 426 DEFeGs and intersected them with the most significant module obtained from the WGCNA analysis. This intersection revealed 17 genes consequentially involved in ferroptosis and RA. Enrichment analysis of these 17 genes showed their primary involvement in the negative regulation of inflammatory mediators and free radicals, response to oxidative stress, negative regulation of the apoptotic process, and the molecular function of identical protein binding. Using machine learning algorithms, we identified the hub genes of RA from these 17 candidate genes. By integrating the top features from LASSO, RF, and SVM, six hub genes- NAMPT, SAT1, FASN, CISD2, LACTB, and PRNP were recognized and these findings suggest that the hub genes not only participate in regulating oxidative stress and inflammation but also serve potentially serve as biomarkers and therapeutic targets for RA.

Nicotinamide phosphoribosyltransferase (NAMPT) is an enzyme involved in the biosynthesis of nicotinamide adenine dinucleotide, an important molecule in the metabolism of cellular energy and maintenance of redox balance. It has key roles in energy homeostasis regulation, immune response, stress response, and cell survival under normal conditions. Dysregulated NAMPT expression has contributed to increased cell sensitization to oxidative stress, and the altered NAD+ metabolism under this environment is an important factor determining the form of cell death [38,39]. Furthermore, the dysregulation of NAMPT expression is reported to be associated with several diseases with inflammation as the major pathological factor, and early research has reported elevated NAMPT levels in patients with rheumatoid arthritis, putting forward it as one of the biomarkers in RA [40]. NAMPT interacts with transcription factors, such as NFKB1, inducing pro-inflammatory mediators like IL6, MMP1, and MMP3 in synovial fibroblasts of RA patients, which may contribute to joint inflammation [41]. Another hub gene identified in our study is Spermidine/ Spermine N1-acetyltransferase 1 (SAT1), located on the X chromosome, producing a coding and a non-coding variant. Previous studies identified p53 as a transcriptional regulator of SAT1, and its involvement in regulating oxidative stress response and ferroptosis [42]. SAT1 induced by p53 activates a downstream target ALOX15 to induce ferroptosis upon stress in the cell due to the accumulation of reactive oxygen species [42,43]. Similarly, it is also reported to have an elevated expression in patients with RA, where it may contribute to oxidative stress and promotion of inflammation [44]. This trend was consistent in our experimental and validation datasets, reinforcing its potential role in RA pathogenesis. A further core gene identified in this exploration is Fatty acid synthase (FASN), located at chromosomal position 17q25.3, and has critical roles in fatty acid and lipid metabolism pathways [45]. FASN is a known contributor to lipid metabolism, potentially by regulating the composition of polyunsaturated fatty acids (PUFAs), a substrate of lipid peroxidation and a hallmark of ferroptosis [46]. Downregulation of FASN has been associated with increased iron accumulation, lipid peroxidation, and ROS production, assisting a sustained inflammation, all of which promote oxidative stress in RA [47]. However, the role of FASN in RA pathogenesis remains controversial, with studies suggesting both therapeutic potential and pathological involvement [48]. Another central gene highlighted in our analysis is the CDGSH iron sulfur domain 2 (CISD2) gene located on chromosome 4q24, encoding a protein localized in the endoplasmic reticulum and the outer membrane of mitochondria. It has a protective function of regulating cellular redox balance and defense against oxidative stress, thereby decelerating ferroptosis [49]. In our study, CISD2 was upregulated in patients with RA, which might indicate a potential compensatory mechanism to counteract mitochondrial dysfunction and associated oxidative stress in RA. However, further experimental validations are required to validate this hypothesis. Another pivotal gene revealed by our findings is LACTB, which has a significant role in lipid metabolism and cell differentiation. Like CISD2, LACTB appears to exhibit protective effects by controlling mitochondrial dysfunction and dynamics. Permuted expression of LACTB in RA may confer increased oxidative stress, inflammation, and deteriorating joint health, though its specific role in RA remains to be elucidated [50,51]. Although there is no direct evidence linking the hub gene PRNP to RA, it has been extensively studied for its role in ferroptotic cell death, oxidative stress modulation, and lipid peroxidation [52,53]. Its potential contribution to RA pathogenesis warrants further investigation. Our correlation analysis revealed strong positive correlations between most hub genes. However, weak negative correlations were observed for the gene pairs CISD2-FASN, LACTB-FASN, and FASN-NAMPT, suggesting distinct functional roles in RA pathogenesis. While these findings establish the mechanistic roles of the hub genes in ferroptosis and oxidative stress, functional annotation analyses further reveal their involvement in key pathways and processes underlying RA pathology.

The functional annotation of our hub genes revealed that CISD2 is particularly involved in autophagy and the regulation of mitochondrial functions, which shows the role of CISD2 in maintaining homeostasis of the cell under stress, potentially affecting the pathways associated with ferroptosis [54]. Its molecular functions, like protein binding, metal ion binding, and RNA binding, also elaborate on these associations of CISD2 to stress response pathways, dysregulation of which could increase the inflammatory process, leading to joint destruction and disease progression [55]. The enrichment of ontology processes like fatty acid metabolism and fatty acid biosynthetic process showcases the role of FASN in lipid metabolism and contributes directly to the accumulation of lipid peroxides, which are significant in ferroptosis [56]. The role of FASN in immune regulation is also highlighted with ontology processes like inflammatory response, neutrophil, and monocyte differentiation [57,48]. The link with osteoblast differentiation potentially shows its influence on bone remodeling, and dysregulation of FASN activity leading to bone erosion, a prominent feature of worsening RA [58,59]. While molecular functions like oxidoreductase activity show their effect on oxidative stress, the cellular components like the Golgi apparatus show their influence in metabolism, suggesting the role of FASN in ferroptotic pathway-mediated amplification of inflammation and joint destruction. Maintaining cellular homeostasis is very important under stress, and the enrichment of the proteolytic process suggests the role of LACTB in protein regulation. Like FASN, LACTB is also involved in lipid metabolic processes, linking the dysregulation of LACTB to the accumulation of lipid peroxides, accelerating ferroptosis, and cellular localization of LACTB to mitochondria and cytosol shows its role in energy metabolism [60]. The gene ontology of NAMPT revealed signal transduction and cell signaling enrichment, signifying its role in response to inflammation and stress. Also, the positive regulation of cell population and proliferation shows its influence on hyperproliferation as seen in synovial fibroblast activation in RA [61]. The presence of NAMPT in the extracellular space and extracellular exosomes potentially shows its role as a pro-inflammatory cytokine, with molecular functions like cytokine activity [40,62]. Further, the KEGG analysis also confirms the above with the enrichment of metabolic pathways and NOD-like receptor signaling pathways, which are linked to the pathological subprocesses of ferroptosis and RA [63]. Though primarily studied in neurological disorders, the functional annotation analysis of PRNP showed potential roles that might have a role in RA. As oxidative stress is an important characteristic of ferroptosis, the response to oxidative stress, enriched for PRNP, orients it with the ferroptotic pathway [53,52]. Also, its role in the negative regulation of type II interferon, IL-17, and IL-2 production, negative regulation of apoptosis, and activated T cell proliferation suggests a potential engagement in immune response modulation and cell survival. Mitochondria and endoplasmic reticulum are central to ferroptosis, yielding a regulation in lipid peroxidation and oxidative stress, and cellular components such as cytoplasm, Golgi apparatus, dendritic cells, including endoplasmic reticulum, and mitochondria were enriched as the localization in cellular components of PRNP. Although the direct link of PRNP to RA is underexplored, its role in oxidative stress and neurological disorders may suggest a potential cross-talk between immune and neuronal pathways. Finally, the role of SAT1 in preserving cellular homeostasis, oxidative response, and regulation of metabolism is shown by the enrichment of biological processes like angiogenesis, polyamine biosynthetic process, putrescine catabolic process, and spermidine acetylation. The dysregulated angiogenesis and metabolic stress are instrumental in synovial hyperplasia and inflammation, and polyamines like spermidine and putrescine are known modulators of oxidative stress [64,65]. The pathway analysis further connects SAT1 to arginine and proline metabolism, metabolic pathways, and ferroptosis, known to influence RA pathogenesis and progression [66,67]. Collectively, the functional annotation and pathway enrichment analyses highlight the diverse roles of our hub genes in ferroptosis, oxidative stress, and immune modulation. This functional diversity suggests that these genes may also play critical roles in regulating immune cell dynamics and to further study this, we explored the correlation of hub genes with immune cell types.

Immune cells play a significant role in the occurrence and progression of RA and ferroptosis, particularly through their function in assisting inflammation, oxidative stress, cytokine production, and cell death [68,69]. Exploring the correlation between the hub genes and immune cells is important for understanding their roles in immune regulation. All the hub genes identified in our study exhibited a strong positive correlation with immune cell enrichment, reflecting their diverse roles in modulating immune responses. CISD2 expression was not limited or specific to a particular set of immune cell types. The extended expression of the gene indicates its generalized role in immune cell mechanisms and might be important for the maintenance of immune homeostasis [54,70]. On the other hand, FASN showed minimal expression in the subset of immune cells in the study. This trend in expression may reflect their tight regulation or activation under very specific stimuli [71]. However, its low expression in selected immune cells, such as T memory cells and myeloid dendritic cells, potentially points towards its role in the functional maintenance and stability of these immune cells. Similar to CISD2, LACTB also showed low immune specificity, showing a prominent enrichment in the innate immune cells, prospectively involved in processes such as antigen-presenting, and regulation of inflammatory response. In contrast, PRNP was specifically found to be enriched in basophils, which are fundamentally involved in allergic response, defense against pathogens, and inflammatory mediator modulation [72]. Though the correlation of PRNP with RA manifestation has been explored the least, basophil enrichment raises possibilities about its function in allergic inflammation and autoimmunity [73]. The hub gene SAT1 was also found to be group-enriched in a subset of innate immune cells, like monocytes. With the known functions of SAT1 in cell death and stress response, enrichment of the gene in the monocyte subset points towards its role in modulating immune activation, shaping the immune microenvironment, and inflammation [74,75]. Additionally, NAMPT appeared to be enriched in neutrophils, another innate immune cell. The enrichment of NAMPT in neutrophils might indicate its significance in assisting the high metabolic condition during the activation to effector function of these cells in inflammation and cell death [76,77]. Together, these findings reveal the integral roles of hub genes in shaping the immune microenvironment and driving the inflammatory processes characteristic of RA.

Through the characterization of the significant genes between RA and ferroptosis, our study proposes important insights into the molecular pathology of ferroptosis in RA. The coregulatory network analysis revealed close associations among hub genes and their regulatory molecules, like TFs and miRNAs, showcasing the complexities involved in RA pathogenesis. All our hub genes showed strong connections with TFs involved in immune response elicitation. Notably, NAMPT was connected to significant regulators like CREB1, NFIL3, and hsa-miR-1, which are all involved in maintaining the integrity of cartilage tissue and modulation of inflammation [78–80]. These regulatory relationships suggest the role of NAMPT in promoting cellular homeostasis and preventing inflammatory damage to joint tissues. A notable finding was the shared regulation of SAT1 and FASN through regulators like hsa-miR-574-5p, a known activator of toll-like receptor 7/8 in mediating increased osteoclast maturation [81,82]. The differential expression of miR-574-5p has been previously associated with inflammation-associated cancers and other autoimmune disorders, suggesting its potential as a biomarker and therapeutic target [82]. This also underscores the potential of these hub genes to act as molecular regulators in crucial pathways like inflammation, ferroptosis, and lipid metabolism, which are intrinsic to the progression of the disease.

**Fig 12:**
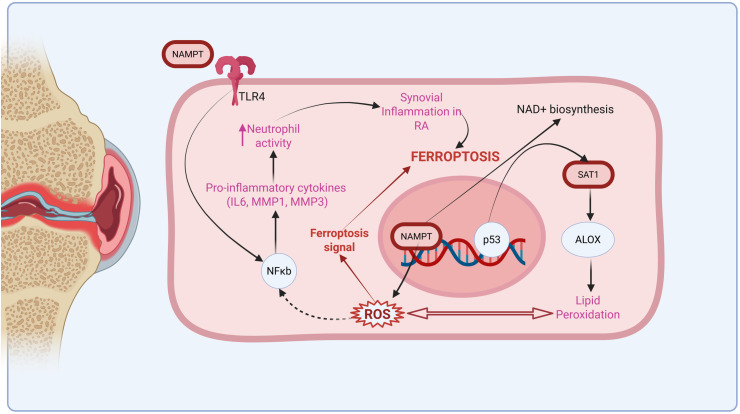
Illustrative insight into the role of NAMPT and SAT1 in ferroptosis and RA pathogenesis.

Conclusively, the robustness of our study findings was validated using an external dataset, reinforcing the robustness of the identified hub genes, particularly NAMPT AND SAT1. The identification of NAMPT and SAT1 as consistently upregulated hub genes across both discovery and validation datasets highlights their potential as clinically actionable biomarkers in RA. Their high diagnostic accuracy (AUC ~ 99%) underscores their utility in distinguishing RA from healthy samples, suggesting strong diagnostic value. NAMPT, in particular, is a key regulator of cellular metabolism and inflammation and has been implicated in several chronic inflammatory diseases, making it a promising candidate for drug repurposing [40]. SAT1, involved in polyamine metabolism, may reflect an underlying metabolic imbalance associated with RA pathogenesis [42]. Given their roles in ferroptosis, a regulated form of cell death linked to inflammation and immune dysfunction, these genes may also serve as potential therapeutic targets. Targeting ferroptosis-related pathways could open avenues for novel therapeutic interventions that go beyond symptom management and directly modulate disease progression. Thus, our findings lay the groundwork for the development of diagnostic and therapeutic strategies in RA, with future studies warranted to validate these markers in larger patient cohorts and explore their mechanistic roles through experimental models. To present our findings as a schematic diagram (Fig 12) showing the roles of NAMPT and SAT1 in regulating ferroptosis within the RA microenvironment [83]. Through interaction with TLR4-mediated NF-κB signalling, NAMPT promotes the release of pro-inflammatory cytokines, such as MMP3, MMP1, and IL-6, further contributing to synovial inflammation and neutrophil activation in RA. Moreover, NAMPT facilitates NAD+ biosynthesis and enhances reactive oxygen species (ROS) generation, which drives oxidative stress and susceptibility to ferroptosis. SAT1, a known p53-regulated gene, accelerates polyamine catabolism and induces the expression of ALOX family enzymes, which also contributes to significant lipid peroxidation. The combined increase in ROS and lipid peroxidation leads to the cellular membrane destabilization, a hallmark of ferroptosis. These interconnected mechanisms strongly suggest that NAMPT and SAT1 may act as key mediators connecting ferroptosis and chronic inflammation in RA, offering insights into disease progression and potential therapeutic targeting.

However, we acknowledge certain limitations in our approach, including the independent transcriptomics analysis and the smaller sample size in the validation dataset, which may have contributed to the lack of significance in some genes, possibly due to dataset heterogeneity. The absence of complete demographic metadata and detailed disease staging introduces potential heterogeneity that could affect expression analysis. Although the RA samples are labelled as early or established, the lack of precise staging criteria limited our ability to conduct stratified analysis. However, to address possible confounding effects from gender differences, we performed gender-stratified analysis of NAMPT and SAT1 expression and observed no significant differences, suggesting minimal bias from this variable. Another notable limitation is the imbalance in sample distribution within the primary dataset, where the number of RA samples exceeded that of the healthy controls. To address this, we employed the SMOTE, which synthetically generates new instances of the minority class to improve the class balance during model training. Despite these efforts, larger and more diverse cohorts, along with in vitro and in vivo functional validation, are essential to confirm the mechanistic roles of the identified genes and strengthen the clinical relevance of these findings.

## 5. Conclusion

Our study demonstrated a significant association between ferroptosis-associated genes and RA, with a strong correlation observed between these genes and immune cell infiltration, providing insight into their functional role in the disease’s pathology. Using a machine learning-based approach, NAMPT, SAT1, FASN, CISD2, PRNP, and LACTB were identified as key genes associated with the occurrence and progression of ferroptosis in RA. Further downstream analyses streamline NAMPT and SAT1 as promising diagnostic biomarkers and therapeutic targets. These findings contribute to the improvement of diagnosis and clinical prognosis in patients with RA.

## Supporting information

S1 File Additional files. (XLSX)

## References

[1] Smolen JS, Aletaha D, McInnes IB. Rheumatoid arthritis. Lancet. 2016;388(10055):2023–38. doi: 10.1016/S0140-6736(16)30173-8 27156434

[2] Wu D, Luo Y, Li T, Zhao X, Lv T, Fang G, et al. Systemic complications of rheumatoid arthritis: Focus on pathogenesis and treatment. Front Immunol. 2022;13:1051082. doi: 10.3389/fimmu.2022.1051082 36618407 PMC9817137

[3] Kim J-W, Suh C-H. Systemic manifestations and complications in patients with rheumatoid arthritis. J Clin Med. 2020;9(6):2008. doi: 10.3390/jcm9062008 32604884 PMC7356332

[4] Boutet M-A, Nerviani A, Fossati-Jimack L, Hands-Greenwood R, Ahmed M, Rivellese F, et al. Comparative analysis of late-stage rheumatoid arthritis and osteoarthritis reveals shared histopathological features. Osteoarthritis Cartilage. 2024;32(2):166–76. doi: 10.1016/j.joca.2023.10.009 37984558

[5] Small A, Lowe K, Wechalekar MD. Immune checkpoints in rheumatoid arthritis: progress and promise. Front Immunol. 2023;14:1285554. doi: 10.3389/fimmu.2023.1285554 38077329 PMC10704353

[6] Weyand CM, Goronzy JJ. The immunology of rheumatoid arthritis. Nat Immunol. 2021;22(1):10–8. doi: 10.1038/s41590-020-00816-x 33257900 PMC8557973

[7] Tong L, Qiu J, Xu Y, Lian S, Xu Y, Wu X. Programmed cell death in rheumatoid arthritis. J Inflamm Res. 2025;18:2377–93. doi: 10.2147/JIR.S499345 39991656 PMC11846511

[8] Shi MZ, Wu QQ, Liu XQ, Guo ZP, Bai YT, Liu YM, et al. Visualising research trends and hotspots in ferroptosis: a decade of insights into iron-dependent cell death and clinical diseases. Discover Life. 2024;54:1–12. doi: 10.1007/S11084-024-09662-5

[9] Li J, Cao F, Yin H-L, Huang Z-J, Lin Z-T, Mao N, et al. Ferroptosis: past, present and future. Cell Death Dis. 2020;11(2):88. doi: 10.1038/s41419-020-2298-2 32015325 PMC6997353

[10] Ma T, Du J, Zhang Y, Wang Y, Wang B, Zhang T. GPX4-independent ferroptosis-a new strategy in disease’s therapy. Cell Death Discov. 2022;8(1):434. doi: 10.1038/s41420-022-01212-0 36309489 PMC9617873

[11] Han C, Liu Y, Dai R, Ismail N, Su W, Li B. Ferroptosis and its potential role in human diseases. Front Pharmacol. 2020;11:239. doi: 10.3389/fphar.2020.00239 32256352 PMC7090218

[12] Zhang S, Xin W, Anderson GJ, Li R, Gao L, Chen S, et al. Double-edge sword roles of iron in driving energy production versus instigating ferroptosis. Cell Death Dis. 2022;13(1):40. doi: 10.1038/s41419-021-04490-1 35013137 PMC8748693

[13] Chen X, Yu C, Kang R, Tang D. Iron metabolism in ferroptosis. Front Cell Dev Biol. 2020;8:590226. doi: 10.3389/FCELL.2020.590226 33117818 PMC7575751

[14] Oh S-J, Ikeda M, Ide T, Hur KY, Lee M-S. Mitochondrial event as an ultimate step in ferroptosis. Cell Death Discov. 2022;8(1):414. doi: 10.1038/s41420-022-01199-8 36209144 PMC9547870

[15] Oh S-J, Ikeda M, Ide T, Hur KY, Lee M-S. Correction: Mitochondrial event as an ultimate step in ferroptosis. Cell Death Discov. 2022;8(1):422. doi: 10.1038/s41420-022-01223-x 36266268 PMC9584973

[16] Zhao T, Yang Q, Xi Y, Xie Z, Shen J, Li Z, et al. Ferroptosis in rheumatoid arthritis: a potential therapeutic strategy. Front Immunol. 2022;13:779585. doi: 10.3389/fimmu.2022.779585 35185879 PMC8847160

[17] Łuczaj W, Gindzienska-Sieskiewicz E, Jarocka-Karpowicz I, Andrisic L, Sierakowski S, Zarkovic N, et al. The onset of lipid peroxidation in rheumatoid arthritis: consequences and monitoring. Free Radic Res. 2016;50(3):304–13. doi: 10.3109/10715762.2015.1112901 26764956

[18] Yadav UCS. Oxidative stress-induced lipid peroxidation: role in inflammation. Free radicals in human health and disease. Springer India; 2014: 119–29. doi: 10.1007/978-81-322-2035-0_9

[19] Zhao H, Tang C, Wang M, Zhao H, Zhu Y. Ferroptosis as an emerging target in rheumatoid arthritis. Front Immunol. 2023;14:1260839. doi: 10.3389/fimmu.2023.1260839 37928554 PMC10620966

[20] Miotto G, Rossetto M, Di Paolo ML, Orian L, Venerando R, Roveri A, et al. Insight into the mechanism of ferroptosis inhibition by ferrostatin-1. Redox Biol. 2020;28:101328. doi: 10.1016/j.redox.2019.101328 31574461 PMC6812032

[21] Guo Y, Walsh AM, Fearon U, Smith MD, Wechalekar MD, Yin X, et al. CD40L-dependent pathway is active at various stages of rheumatoid arthritis disease progression. J Immunol. 2017;198(11):4490–501. doi: 10.4049/jimmunol.1601988 28455435

[22] Lin Y, He J, Mou Z, Chen H, You W, Guan T, et al. Ferroptosis-related genes, a novel therapeutic target for focal segmental glomerulosclerosis. BMC Nephrol. 2024;25(1):58. doi: 10.1186/s12882-024-03490-5 38368317 PMC10874534

[23] Love MI, Huber W, Anders S. Moderated estimation of fold change and dispersion for RNA-seq data with DESeq2. Genome Biol. 2014;15(12):550. doi: 10.1186/s13059-014-0550-8 25516281 PMC4302049

[24] Wickham H. ggplot2. Springer New York; 2009. doi: 10.1007/978-0-387-98141-3

[25] Langfelder P, Horvath S. WGCNA: an R package for weighted correlation network analysis. BMC Bioinform. 2008;9:559. doi: 10.1186/1471-2105-9-559 19114008 PMC2631488

[26] Li J, Zhou D, Qiu W, Shi Y, Yang J-J, Chen S, et al. Application of weighted gene co-expression network analysis for data from paired design. Sci Rep. 2018;8(1):622. doi: 10.1038/s41598-017-18705-z 29330528 PMC5766625

[27] Shannon P, Markiel A, Ozier O, Baliga NS, Wang JT, Ramage D, et al. Cytoscape: a software environment for integrated models of biomolecular interaction networks. Genome Res. 2003;13(11):2498–504. doi: 10.1101/gr.1239303 14597658 PMC403769

[28] Mongardi S, Cascianelli S, Masseroli M. Biologically weighted LASSO: enhancing functional interpretability in gene expression data analysis. Bioinformatics. 2024;40(10):btae605. doi: 10.1093/bioinformatics/btae605 39412436 PMC11639179

[29] Tadist K, Najah S, Nikolov NS, Mrabti F, Zahi A. Feature selection methods and genomic big data: a systematic review. J Big Data. 2019;6:1–24. doi: 10.1186/S40537-019-0241-0

[30] Cheng Y, Xu S-M, Santucci K, Lindner G, Janitz M. Machine learning and related approaches in transcriptomics. Biochem Biophys Res Commun. 2024;724:150225. doi: 10.1016/j.bbrc.2024.150225 38852503

[31] Singh VK, Singh MP. Predicting inhibitor development in hemophilia “A” using machine learning: a comprehensive approach to data preprocessing, balancing, and biomarker identification using AI on the CHAMP dataset. Curr Pharm Biotechnol. 2025:10.2174/0113892010366485250415101928. doi: 10.2174/0113892010366485250415101928 40264326

[32] Sahoo K, Sundararajan V. IL-1β and associated molecules as prognostic biomarkers linked with immune cell infiltration in colorectal cancer: an integrated statistical and machine learning approach. Discov Oncol. 2025;16(1):252. doi: 10.1007/s12672-025-01989-3 40019680 PMC11871282

[33] Li K, Li Y, Gao Q, Xu L, Hu Q, Ji B, et al. Machine learning in risk prediction of continuous renal replacement therapy after surgical repair of acute type A aortic dissection. J Cardiothorac Vasc Anesth. 2025;39(10):2739–47. doi: 10.1053/j.jvca.2025.06.028 40645830

[34] Robin X, Turck N, Hainard A, Tiberti N, Lisacek F, Sanchez J-C, et al. pROC: an open-source package for R and S+ to analyze and compare ROC curves. BMC Bioinform. 2011;12:77. doi: 10.1186/1471-2105-12-77 21414208 PMC3068975

[35] Li X, He A, Liu Y, Huang Y, Zhang X. Bioinformatics identification of ferroptosis-related genes and therapeutic drugs in rheumatoid arthritis. Front Med (Lausanne). 2023;10:1192153. doi: 10.3389/fmed.2023.1192153 37521346 PMC10374025

[36] Zhou G, Soufan O, Ewald J, Hancock REW, Basu N, Xia J. NetworkAnalyst 3.0: a visual analytics platform for comprehensive gene expression profiling and meta-analysis. Nucleic Acids Res. 2019;47(W1):W234–41. doi: 10.1093/nar/gkz240 30931480 PMC6602507

[37] Zhu R, Gao C, Feng Q, Guan H, Wu J, Samant H, et al. Ferroptosis-related genes with post-transcriptional regulation mechanisms in hepatocellular carcinoma determined by bioinformatics and experimental validation. Ann Transl Med. 2022;10(24):1390. doi: 10.21037/atm-22-5750 36660631 PMC9843431

[38] Nishida T, Naguro I, Ichijo H. NAMPT-dependent NAD+ salvage is crucial for the decision between apoptotic and necrotic cell death under oxidative stress. Cell Death Discov. 2022;8(1):195. doi: 10.1038/s41420-022-01007-3 35410407 PMC9001718

[39] Fan J, Zhu T, Tian X, Liu S, Zhang S-L. Exploration of ferroptosis and necroptosis-related genes and potential molecular mechanisms in psoriasis and atherosclerosis. Front Immunol. 2024;15:1372303. doi: 10.3389/fimmu.2024.1372303 39072329 PMC11272566

[40] Cheng X-P, Wang X-W, Sun H-F, Xu L, Olatunji OJ, Li Y, et al. NAMPT/SIRT1 expression levels in white blood cells differentiate the different rheumatoid arthritis subsets: an inspiration from traditional Chinese medicine. J Inflamm Res. 2023;16:4271–85. doi: 10.2147/JIR.S431600 37791116 PMC10543492

[41] Brentano F, Schorr O, Ospelt C, Stanczyk J, Gay RE, Gay S, et al. Pre-B cell colony-enhancing factor/visfatin, a new marker of inflammation in rheumatoid arthritis with proinflammatory and matrix-degrading activities. Arthritis Rheum. 2007;56(9):2829–39. doi: 10.1002/art.22833 17763446

[42] Ou Y, Wang S-J, Li D, Chu B, Gu W. Activation of SAT1 engages polyamine metabolism with p53-mediated ferroptotic responses. Proc Natl Acad Sci U S A. 2016;113(44):E6806–12. doi: 10.1073/pnas.1607152113 27698118 PMC5098629

[43] Kang R, Kroemer G, Tang D. The tumor suppressor protein p53 and the ferroptosis network. Free Radic Biol Med. 2019;133:162–8. doi: 10.1016/j.freeradbiomed.2018.05.074 29800655 PMC6251771

[44] Neidhart M, Karouzakis E, Jüngel A, Gay RE, Gay S. Inhibition of spermidine/spermine N1-acetyltransferase activity: a new therapeutic concept in rheumatoid arthritis. Arthritis Rheumatol. 2014;66(7):1723–33. doi: 10.1002/art.38574 24578214

[45] Locke GA, Cheng D, Witmer MR, Tamura JK, Haque T, Carney RF, et al. Differential activation of recombinant human acetyl-CoA carboxylases 1 and 2 by citrate. Arch Biochem Biophys. 2008;475(1):72–9. doi: 10.1016/j.abb.2008.04.011 18455495

[46] Liang D, Minikes AM, Jiang X. Ferroptosis at the intersection of lipid metabolism and cellular signaling. Mol Cell. 2022;82(12):2215–27. doi: 10.1016/j.molcel.2022.03.022 35390277 PMC9233073

[47] Li Y, Yang W, Zheng Y, Dai W, Ji J, Wu L, et al. Targeting fatty acid synthase modulates sensitivity of hepatocellular carcinoma to sorafenib via ferroptosis. J Exp Clin Cancer Res. 2023;42(1):6. doi: 10.1186/s13046-022-02567-z 36604718 PMC9817350

[48] Xiao Y, Yang Y, Xiong H, Dong G. The implications of FASN in immune cell biology and related diseases. Cell Death Dis. 2024;15(1):88. doi: 10.1038/s41419-024-06463-6 38272906 PMC10810964

[49] Li Y, Xu B, Ren X, Wang L, Xu Y, Zhao Y, et al. Inhibition of CISD2 promotes ferroptosis through ferritinophagy-mediated ferritin turnover and regulation of p62–Keap1–NRF2 pathway. Cell Mol Biol Lett. 2022;27:1–18. doi: 10.1186/S11658-022-00383-Z 36180832 PMC9523958

[50] Cui L, Weiyao J, Chenghong S, Limei L, Xinghua Z, Bo Y, et al. Rheumatoid arthritis and mitochondrial homeostasis: The crossroads of metabolism and immunity. Front Med (Lausanne). 2022;9:1017650. doi: 10.3389/fmed.2022.1017650 36213670 PMC9542797

[51] Zeng K, Huang N, Liu N, Deng X, Mu Y, Zhang X, et al. LACTB suppresses liver cancer progression through regulation of ferroptosis. Redox Biol. 2024;75:103270. doi: 10.1016/j.redox.2024.103270 39047638 PMC11321384

[52] Lin C, He J, Tong X, Song L. Copper homeostasis-associated gene PRNP regulates ferroptosis and immune infiltration in breast cancer. PLoS One. 2023;18(8):e0288091. doi: 10.1371/journal.pone.0288091 37535656 PMC10399738

[53] Xu Y, Hao J, Chen Q, Qin Y, Qin H, Ren S, et al. Inhibition of the RBMS1/PRNP axis improves ferroptosis resistance-mediated oxaliplatin chemoresistance in colorectal cancer. Mol Carcinog. 2024;63(2):224–37. doi: 10.1002/mc.23647 37861356

[54] Shen Z-Q, Huang Y-L, Teng Y-C, Wang T-W, Kao C-H, Yeh C-H, et al. CISD2 maintains cellular homeostasis. Biochim Biophys Acta Mol Cell Res. 2021;1868(4):118954. doi: 10.1016/j.bbamcr.2021.118954 33422617

[55] Lin C-C, Chiang T-H, Sun Y-Y, Lin M-S. Protective effects of CISD2 and influence of curcumin on CISD2 expression in aged animals and inflammatory cell model. Nutrients. 2019;11(3):700. doi: 10.3390/nu11030700 30934593 PMC6470567

[56] Fhu CW, Ali A. Fatty acid synthase: an emerging target in cancer. Molecules. 2020;25(17):3935. doi: 10.3390/molecules25173935 32872164 PMC7504791

[57] Xiong Q, Feng D, Wang Z, Ying Y, Xu C, Wei Q, et al. fatty acid synthase is the key regulator of fatty acid metabolism and is related to immunotherapy in bladder cancer. Front Immunol. 2022;13:836939. doi: 10.3389/fimmu.2022.836939 35392075 PMC8982515

[58] Chen C-Y, Tseng K-Y, Wong Z-H, Chen Y-P, Chen T-Y, Chen H-Y, et al. Cooperative impact of thiazolidinedione and fatty acid synthase on human osteogenesis. Aging (Albany NY). 2019;11(8):2327–42. doi: 10.18632/aging.101916 31005954 PMC6519991

[59] Bermeo S, Al Saedi A, Vidal C, Khalil M, Pang M, Troen BR, et al. Treatment with an inhibitor of fatty acid synthase attenuates bone loss in ovariectomized mice. Bone. 2019;122:114–22. doi: 10.1016/j.bone.2019.02.017 30779961

[60] Zhang M, Wu B, Gu J. The pivotal role of LACTB in the process of cancer development. Int J Mol Sci. 2025;26(3):1279. doi: 10.3390/ijms26031279 39941048 PMC11818536

[61] Mercurio L, Morelli M, Scarponi C, Scaglione GL, Pallotta S, Avitabile D, et al. Enhanced NAMPT-mediated NAD salvage pathway contributes to psoriasis pathogenesis by amplifying epithelial auto-inflammatory circuits. Int J Mol Sci. 2021;22(13):6860. doi: 10.3390/ijms22136860 34202251 PMC8267663

[62] Garten A, Petzold S, Schuster S, Körner A, Kratzsch J, Kiess W. Nampt and its potential role in inflammation and type 2 diabetes. Handb Exp Pharmacol. 2011;(203):147–64. doi: 10.1007/978-3-642-17214-4_7 21484571

[63] Madahar SS, Gideon A, Abdul-Sater AA. Nod-like receptors in inflammatory arthritis. Biomed J. 2024;47(1):100655. doi: 10.1016/j.bj.2023.100655 37598797 PMC10825342

[64] Giordo R, Posadino AM, Maccioccu P, Capobianco G, Zinellu A, Erre GL, et al. Sera from rheumatoid arthritis patients induce oxidative stress and pro-angiogenic and profibrotic phenotypes in human endothelial cells. J Clin Med. 2024;13(19):5913. doi: 10.3390/jcm13195913 39407973 PMC11477295

[65] Ueno D, Ikeda K, Yamazaki E, Katayama A, Urata R, Matoba S. Spermidine improves angiogenic capacity of senescent endothelial cells, and enhances ischemia-induced neovascularization in aged mice. Sci Rep. 2023;13(1):8338. doi: 10.1038/s41598-023-35447-3 37221395 PMC10205711

[66] Xu L, Chang C, Jiang P, Wei K, Zhang R, Jin Y, et al. Metabolomics in rheumatoid arthritis: advances and review. Front Immunol. 2022;13:961708. doi: 10.3389/FIMMU.2022.961708 36032122 PMC9404373

[67] Vettore LA, Westbrook RL, Tennant DA. Proline metabolism and redox; maintaining a balance in health and disease. Amino Acids. 2021;53(12):1779–88. doi: 10.1007/s00726-021-03051-2 34291343 PMC8651533

[68] Dang Q, Sun Z, Wang Y, Wang L, Liu Z, Han X. Ferroptosis: a double-edged sword mediating immune tolerance of cancer. Cell Death Dis. 2022;13(11):925. doi: 10.1038/s41419-022-05384-6 36335094 PMC9637147

[69] Jang S, Kwon E-J, Lee JJ. Rheumatoid arthritis: pathogenic roles of diverse immune cells. Int J Mol Sci. 2022;23(2):905. doi: 10.3390/ijms23020905 35055087 PMC8780115

[70] Choi UY, Choi YJ, Lee S-A, Yoo J-S. Cisd2 deficiency impairs neutrophil function by regulating calcium homeostasis via Calnexin and SERCA. BMB Rep. 2024;57(5):256–61. doi: 10.5483/BMBRep.2024-0011 38627949 PMC11139677

[71] Wang K, Li L, Jin J, An Y, Wang Z, Zhou S, et al. Fatty acid synthase (Fasn) inhibits the expression levels of immune response genes via alteration of alternative splicing in islet cells. J Diabetes Complications. 2022;36(6):108159. doi: 10.1016/j.jdiacomp.2022.108159 35210136

[72] Lathe R, Darlix J-L. Prion protein PRNP: a new player in innate immunity? The Aβ connection. J Alzheimers Dis Rep. 2017;1(1):263–75. doi: 10.3233/ADR-170037 30480243 PMC6159716

[73] Sharma M, Bayry J. Autoimmunity: Basophils in autoimmune and inflammatory diseases. Nat Rev Rheumatol. 2015;11(3):129–31. doi: 10.1038/nrrheum.2014.199 25422004

[74] Mou Y, Zhang L, Liu Z, Song X. Abundant expression of ferroptosis-related SAT1 is related to unfavorable outcome and immune cell infiltration in low-grade glioma. BMC Cancer. 2022;22(1):215. doi: 10.1186/s12885-022-09313-w 35227235 PMC8883632

[75] Jin Z, Xu H, Sun X, Yan B, Wang L. Targeting SAT1 prevents osteoporosis through promoting osteoclast apoptosis. Biomed Pharmacother. 2024;175:116732. doi: 10.1016/j.biopha.2024.116732 38739990

[76] Peng A, Li J, Xing J, Yao Y, Niu X, Zhang K. The function of nicotinamide phosphoribosyl transferase (NAMPT) and its role in diseases. Front Mol Biosci. 2024;11:1480617. doi: 10.3389/fmolb.2024.1480617 39513038 PMC11540786

[77] Siakaeva E, Pylaeva E, Spyra I, Bordbari S, Höing B, Kürten C, et al. Neutrophil maturation and survival is controlled by IFN-dependent regulation of NAMPT signaling. Int J Mol Sci. 2019;20(22):5584. doi: 10.3390/ijms20225584 31717318 PMC6888478

[78] Du J, Zheng L, Chen S, Wang N, Pu X, Yu D, et al. NFIL3 and its immunoregulatory role in rheumatoid arthritis patients. Front Immunol. 2022;13:950144. doi: 10.3389/fimmu.2022.950144 36439145 PMC9692021

[79] Li G, Han N, Li Z, Lu Q. Identification of transcription regulatory relationships in rheumatoid arthritis and osteoarthritis. Clin Rheumatol. 2013;32(5):609–15. doi: 10.1007/s10067-012-2143-9 23296645

[80] Mirzamohammadi F, Papaioannou G, Kobayashi T. MicroRNAs in cartilage development, homeostasis, and disease. Curr Osteoporos Rep. 2014;12(4):410–9. doi: 10.1007/s11914-014-0229-9 25091054 PMC4234170

[81] Yang JY. miR-574-5p in epigenetic regulation and Toll-like receptor signaling. Cell Commun Signal. 2024;22(1):567. doi: 10.1186/s12964-024-01934-x 39593070 PMC11600836

[82] Hegewald AB, Breitwieser K, Ottinger SM, Mobarrez F, Korotkova M, Rethi B, et al. Extracellular miR-574-5p induces osteoclast differentiation via TLR 7/8 in rheumatoid arthritis. Front Immunol. 2020;11:585282. doi: 10.3389/FIMMU.2020.585282 33154755 PMC7591713

[83] BioRender. BioRender. 2024. https://www.biorender.com/

